# Translation and validation of modified dental anxiety scale based on adult Taiwan population

**DOI:** 10.1186/s12903-021-02017-w

**Published:** 2021-12-17

**Authors:** Chia-Shu Lin, Chen-Yi Lee, Shih-Yun Wu, Li-Ling Chen, Kun-Tsung Lee, Min-Ching Wang, Tze-Fang Wang

**Affiliations:** 1 Department of Dentistry, College of Dentistry, National Yang Ming Chiao Tung University, No. 155, Sec. 2, Linong Street, Taipei, 11221 Taiwan, ROC; 2 Institute of Brain Science, National Yang Ming Chiao Tung University, Hsinchu, Taiwan; 3 Brain Research Center, National Yang Ming Chiao Tung University, Hsinchu, Taiwan; 4 Department of Oral Hygiene, College of Dental Medicine, Kaohsiung Medical University, Kaohsiung, Taiwan; 5 Department of Medical Research, Kaohsiung Medical University Hospital, Kaohsiung, Taiwan; 6 Division of Family Dentistry, Department of Stomatology, Taipei Veterans General Hospital, Taipei, Taiwan; 7 Division of Clinical Dentistry, Department of Dentistry, Kaohsiung Medical University Hospital, Kaohsiung, Taiwan; 8 Department of Dentistry, Taipei Municipal Wanfang Hospital, Taipei, Taiwan; 9 Department of Stomatology, Taipei Veterans General Hospital, Taipei, Taiwan; 10 Department of Nursing, College of Nursing, National Yang Ming Chiao Tung University, Hsinchu, Taiwan

**Keywords:** Dental anxiety, Fear, Pain, Avoidance

## Abstract

**Background:**

Dental anxiety is associated with negative experiences of dental treatment and dental-visiting behavior. The Modified Dental Anxiety Scale (MDAS) is widely used for assessing dental anxiety. The study aims to establish the psychometric properties of a Chinese version of the MDAS based on the Taiwan sample (i.e., T-MDAS).

**Methods:**

The T-MDAS and dental-visiting behavior and experience were assessed for 402 adult subjects recruited from community and clinical sites. The following psychometric properties were assessed: (a) internal consistency, (b) temporal stability, (c) criterion-related validity (i.e., the association with the score of Index of Dental Anxiety and Fear, IDAF-4C), (d) discrimination validity (i.e., the difference in scores between the subjects with and without a habit of a regular dental visit, and (e) the construct validity from a confirmatory factor analysis (CFA). Results. The T-MDAS showed good internal consistency (Cronbach’s α = 0.88) and temporal stability (ρ = 0.69, *p* < 0.001). The score was significantly correlated with the score of the IDAF-4C (ρ = 0.76, *p* < 0.001) and differed between subjects who regularly visited a dentist or not, supporting good criterion-related validity and discrimination validity. Results from CFA supports good construct validity. Furthermore, higher dental anxiety was related to the lack of a regular dental visit, feeling pain during treatment, and feeling insufficient skills and empathy of dentists. A higher proportion of high-dental anxiety subjects in female subjects (8.5%), compared to male subjects (5.0%), was noted.

**Conclusions:**

The T-MDAS is a valid tool for assessing adult dental anxiety. The score is highly associated with dental-visiting behavior and experience of dental patients.

**Supplementary Information:**

The online version contains supplementary material available at 10.1186/s12903-021-02017-w.

## Introduction

Dental anxiety is broadly defined as the anxiety related to dental care, which includes both emotional and cognitive responses to dental treatment [1]. High dental anxiety is markedly associated with the negative experience of dental treatment and behavior of visiting dentists. For example, a meta-analysis of clinical studies revealed that higher dental anxiety is associated with higher state anxiety and pain across different dental procedures [2]. High dental anxiety is strongly associated with avoidance or delay of dental treatment [3–5], which in turn exacerbates patients’ oral disease and leads to a ‘vicious cycle’ of oral health [3]. Moreover, there is a statistically significant association between individuals with higher dental anxiety and the risk of dental phobia, a disorder of specific phobia [6]. The prevalence of high dental anxiety is remarkably high in adults, ranging from 6 to 12%, according to previous surveys [7–9]. Therefore, high dental anxiety in the adult population has been widely considered a major challenge in dental treatment and oral public health.

While different approaches have been proposed for managing patients with high dental anxiety [10, 11], the fundamental issue of managing dental anxiety is to assess individual dental anxiety with a valid and reliable tool. Assessment of dental anxiety has been recommended for all dental patients to screen for patients with high dental anxiety, so that their experience of treatment, can be improved [12]. A variety of tools for assessing dental anxiety, dated back to the four-item Dental Anxiety Scale (DAS) [13]. Extending the original DAS, Humphris et al. developed the Modified Dental Anxiety Scale (MDAS), which consists of five questions of two domains of dental treatment [14]. The two domains include the Anticipatory Dental Anxiety, which relates to the feeling when individuals are about to receive dental treatment, and the Treatment Dental Anxiety, which relates to the feeling when individuals are receiving a specific dental procedure (e.g., scaling or an injection of local anesthetics) [8]. With good validity, reliability, and simplicity (i.e., only five questions), the MDAS has been translated into multiple languages and widely used for the clinical assessment of dental anxiety in the world [15–20].

Dental anxiety is associated with several factors intrinsic to dental treatment. For example, a worse experience of dental treatment (including pain) and a poor patient-dentist relationship may contribute to dental anxiety and fear [21, 22]. However, the association between dental anxiety and the ‘extrinsic factors’ of dental treatment, including the approachability of dental service and the financial burden of treatment, has remained unclear. In Taiwan, most procedures of preventing and treating oral diseases, including caries restoration, ultrasound scaling, extraction, and root canal treatment, are covered by the system of National Health Insurance (NHI) [23]. In the meantime, there is an abundant supply of dental manpower [24], especially for local dental clinics in urban areas. All the factors reduce the barrier to a regular dental visit. Therefore, investigating the association between dental anxiety and dental-visiting behavior and experience in Taiwan may provide new insights into the extrinsic factors of dental anxiety.

The current study has three major research aims. First, we reported the psychometric properties (i.e., criterion-related validity, discrimination validity, construct validity, internal consistency, and temporal stability) of a Chinese version of the MDAS based on the Taiwan sample (i.e., T-MDAS). Second, we investigated the association between dental anxiety and dental-visiting behavior and experience. Additionally, we estimated the proportion of high-dental anxiety individuals, based on the results of the T-MDAS.

## Materials and methods

### Study design

The study is a cross-sectional and observational research focusing on the psychometric properties of the T-MDAS and dental-visiting behavior and experience of the adult Taiwan population. The subjects were recruited from community and clinical sites. The following psychometric properties of the T-MDAS were assessed: (a) internal consistency, (b) temporal stability, (c) criterion-related validity (i.e., the association with the score of Index of Dental Anxiety and Fear, IDAF-4C) [25],

### Participants

The psychometric properties of the T-MDAS were evaluated based on the samples from both a community and a clinical site. For the community sample (n = 201), subjects were recruited via advertisements posted in the local community and the university campus of National Yang-Ming University. For the clinical sample, subjects were recruited in the outpatient department of the Department of Stomatology, Taipei Veterans General Hospital (n = 201). We recruited the subjects with the following inclusion criteria: (a) aged between 20 and 90 years and (b) being able to communicate with the experimenters verbally, and the following exclusion criteria: (a) having a history of major physical or psychiatric disorders and (b) feeling stressed when completing the questionnaires related to fear and anxiety of dental treatment. The same study sample also participated in another study regarding negative experience of dental treatment, which results are published in a previous study [26]. All the questionnaires were collected by the same researcher (L–L Chen). The study was approved by the institutional review board (IRB) of National Yang-Ming University (YM106095E) and the IRB of Taipei Veterans General Hospital (2018–12-003AC). All the subjects completed a written informed consent before the study started. The study is conducted in accordance with the Declaration of Helsinki.

### Estimation of sample size

The sample size of the study is estimated using G*Power ver. 3.1.2 [27] with the following conditions. (a) We hypothesized that the T-MDAS score would discriminate between the subjects with and without a habit of a regular dental visit, as evidence of the validity of the T-MDAS. Therefore, the Mann–Whitney U test was adopted for hypothesis testing. (b) An analysis of statistical power was performed by controlling α = 0.05 and power = 0.85 (i.e., β = 0.15), with a moderate effect size (d = 0.45). Based on the calculation, we estimated the minimal sample size as 188 for each site of the sample (community or clinics) and 376 for both sites of the sample.

### Assessment tools

#### Preparation of the Taiwan MDAS (T-MDAS)

The original English version of the MDAS developed by Humphris et al. [14] was translated by a dentist (K-T Lee) to Traditional Chinese. The Chinese version was back-translated into English and validated independently by another dentist (C-S Lin). The quality of the translation was then independently assessed by a pedodontist (M-C Wang) for expert opinions, respectively for each of the five questions. The assessment showed a good quality of translation (mean point = 4.6) based on a 1–5 five-point numerical scale (1 = Very poor quality and 5 = Very good quality).

#### The index of dental anxiety and fear (IDAF-4C)

The IDAF-4C consists of eight questions that assess the emotional, cognitive, behavioral, and physiological aspects related to dental anxiety and fear [25]. A Chinese version has been previously translated from the original English version and demonstrates a good clinical validity, based on Taiwanese subjects who received extraction of wisdom teeth [28]. In the current study, we adopted the score from the IDAF-4C as the criterion for evaluating the criterion-related validity of the T-MDAS. The scores from the two scales have revealed a high correlation in previous research [29].

#### Dental-visiting behavior and experience

The behavior and experience related to dental visiting were assessed using customized questions. Five variables were collected via the following questions: (a)’How do you think about your oral function?’ (Perceived Oral Function), (b) ‘When you feel something uncomfortable about your mouth, teeth, or gum, what would you do first? (Choices of Oral Care), (c) ‘When was the last time when you visit a dentist? (Latest Visit), (d) ‘Have you had any unpleasant experience about visiting a dentist? (Negative Experience with Dentists), and (e) ‘Do you regularly visit a dentist’ (Regular Dental Visits). The variables ‘Choices of Oral Care’ and ‘Negative Experience with Dentists’ consist of multiple choices. Subjects were instructed to choose all the items they agreed. See Table 1 for the response items of each question.

**Table 1:** Results of descriptive analysis

	n	(%)	Mean	Median	S.D	Min	Max
*Gender*							
Male	202	(50.2)					
Female	200	(49.8)					
Age^1^			47.0	47.0	16.5	20.0	86.0
*Site*							
Community	201	(50.0)					
Clinical	201	(50.0)					
T-MDAS^1^			10.6	10.0	4.3	5.0	25.0
IDAF-4C^1^			1.8	1.4	0.9	1.0	5.0
*Regular dental visits*							
No	167	(41.5)					
Yes	235	(58.5)					
*Perceived oral function*							
Very good	25	(6.2)					
Good	84	(20.9)					
Moderate	217	(54.0)					
Poor	56	(13.9)					
Very poor	20	(5.0)					
*Choices of oral care*^2^							
Visiting a dentist	354	(88.1)					
Topical medication	18	(4.5)					
Taking analgesics	37	(9.2)					
Ignoring it	56	(13.9)					
Others	8	(2.0)					
*Latest visit*							
Within 6 months	266	(66.2)					
6 months–2 years	88	(21.9)					
More than 2 years	48	(11.9)					
*Negative experience with dentists*^2^							
Never visiting a dentist	1	(0.2)					
No negative experience	258	(64.2)					
Pain during treatment	71	(17.7)					
Insufficient skills	69	(17.2)					
Insufficient empathy	41	(10.2)					
Poor communication	33	(8.2)					
Others	37	(9.2)					

^1^The scores are non-normally distributed, based on the Shapiro–Wilk test (*p* < 0.05)

^2^Subjects are allowed to choose more than one item

Max.: maximum, Min.: minimum, IDAF-4C: index of dental anxiety and fear, S.D.: standard deviation, T-MDAS: the Chinese version of the modified dental anxiety scale based on the Taiwanese sample

### Statistical analysis

We first examined the normality of the score distribution of the T-MDAS score and the IDAF-4C score. All the scores are non-normally distributed, based on the Shapiro–Wilk test (*p* < 0.05) (Table 1). Therefore, non-parametric tests were used for the statistical analysis. All the statistical analysis was performed using IBM SPSS Statistics (ver. 24.0) (IBM, Armonk, NY, USA), except for the confirmatory factor analysis (CFA), which was performed using LISREL (ver. 10.20) (Scientific Software International, Inc., Lincolnwood, IL, USA).

#### Analysis of Reliability of the T-MDAS

For the five questions of the T-MDAS, internal consistency was assessed using Cronbach’s alpha. For assessing temporal stability, 30 subjects were asked to perform the same questionnaires again after the first assessment, with a delay of five to six weeks (mean = 5.7 weeks). The strength of association between the test and re-test scores was assessed using Spearman’s rho coefficient and the difference between the two tests was assessed using the Wilcoxon Signed-rank test. Additionally, the intraclass correlation coefficient (ICC) was calculated to assess the agreement and consistency between the test and re-test scores.

#### Analysis of Validity of the T-MDAS

We first investigated the criterion-related validity by assessing the strength of association between the T-MDAS score and the IDAF-4C score (as the criterion), using Spearman’s rho coefficient. Second, we performed an analysis on the discrimination validity of the T-MDAS. Because dental anxiety is highly associated with patient behavior of dental visiting [3, 4], we hypothesized that the T-MDAS score would discriminate between the subjects with and without a habit of a regular dental visit. The difference was assessed using the Mann–Whitney U test. In addition, we performed a CFA to assess the construct validity of the T-MDAS [30]. We tested the two-factor model that differentiates anticipatory dental anxiety and treatment dental anxiety [8] and assessed the overall model fit. The following indices were evaluated: the comparative fit index (CFI), the goodness of fit index (GFI), the normed fit index (NFI), and the root mean square error of approximation (RMSEA).

#### Association between dental anxiety and dental-visiting behavior and experience

We focused on the following variables of dental-visiting behavior and experience, as defined in the previous section: (a) Perceived Oral Function, (b) Choices of Oral Care, (c) Latest Visit, and (d) Negative Experience with Dentists. For the variable Perceived Oral Function, the Kruskal Wallis test was performed for assessing the difference in the T-MDAS score between subjects who reported ‘Very Good’, ‘Good’, ‘Moderate’, ‘Poor’, and ‘Very Poor’ oral functions. For the variable Latest Visit, the Kruskal Wallis test was performed for assessing the difference in the T-MDAS score between subjects who had their last visit ‘Within 6 months’, ‘6 months-2 years’, and ‘More than 2 years’. For the variables Choices of Oral Care and Negative Experience with Dentists, the Mann–Whitney U test was performed, respectively for comparing the T-MDAS score between the subjects who chose and who did not choose each response item of the variables. For example, in Choices of Oral Care, a comparison was made between the subjects who took analgesics for oral care and those who did not. And in Negative Experience with Dentists, a comparison was made between the subjects who felt insufficient skills of dentists and those who did not.

#### The proportion of high-dental anxiety individuals

To estimate the proportion of high-dental anxiety individuals from our samples, we adopted the cut-off value (19 points) for high-dental anxiety, which is established by previous studies based on a U.K. sample [6, 7]. We first calculated the distribution of the T-MDAS score from our sample. And the proportion of high-dental anxiety individuals was calculated according to the cumulating distribution of the score. Notably, because the T-MDAS score was associated with gender (Table 2), the analysis was performed separately for female and male subgroups.

**Table 2:** Results of the comparison between genders and sampling sites

	Community (n = 201)			Female (n = 200)		
	Age	MDAS	IDAF-4C	Age	MDAS	IDAF-4C
Mean	45.5	10.5	1.8	47.0	11.3	1.8
Median	46.0	10.0	1.4	47.0	11.0	1.5
S.D	17.1	4.3	0.9	16.4	4.4	0.9
Min	20.0	5.0	1.0	20.0	5.0	1.0
Max	86.0	25.0	5.0	86.0	23.0	4.8

	Clinical (n = 201)			Male (n = 202)		
Mean	48.5	10.8	1.7	47.0	10.0	1.7
Median	48.0	10.0	1.4	47.5	9.0	1.4
S.D	15.8	4.4	0.9	16.6	4.1	0.9
Min	20.0	5.0	1.0	20.0	5.0	1.0
Max	86.0	25.0	5.0	86.0	25.0	5.0
Comparison^1^	0.072	0.642	0.365	0.976	0.002	0.402

^1^The number denotes the *p* value of Mann–Whitney U test

Max.: maximum, Min.: minimum, IDAF-4C: index of dental anxiety and fear, S.D.: standard deviation, T-MDAS: the Chinese version of the modified dental anxiety scale based on the Taiwanese sample

## Results

### Results of descriptive analysis

Table 1 shows the results of descriptive analysis, including the analysis of age, the T-MDAS score, the IDAF-4C score, and dental-visiting behavior and experience. The comparison between age, the T-MDAS score, and the IDAF-4C score was performed between subjects of different genders and subjects’ samples from the community vs. the clinical sites. As shown in Table 2, no significant difference is found for subjects’ age, the T-MDAS score, and the IDAF-C score, between the community and the clinical samples. Therefore, data from the two samples were combined for the subsequent analyses. The female subjects showed a higher T-MDAS score, compared to the male subjects (two-tailed Mann–Whitney U test, *p* = 0.002) (Table 2).

### Reliability of T-MDAS

The T-MDAS reveals good internal consistency (Cronbach’s α = 0.88). Within the T-MDAS, the scores from each pair of the five questions were significantly correlated (Table 3). For the 30 subjects who completed a test and a re-test, their T-MDAS scores between the two tests were significantly correlated (ρ = 0.69, *p* < 0.001). The test and re-test scores were not significantly different (two-tailed Wilcoxon signed-rank test, *p* > 0.05). An analysis of the ICC revealed that, for the two-factor mixed model, the test and re-test scores showed good absolute agreement (ICC = 0.89) and consistency (ICC = 0.89). The results suggest that the T-MDAS shows good temporal stability within a period of around six weeks.

**Table 3:** Correlation between the questions of the T-MDAS and IDAF-4C

	Questions of the T-MDAS					IDAF-4C
	1	2	3	4	5	
1		0.76	0.60	0.48	0.45	0.68
2			0.68	0.52	0.50	0.69
3				0.56	0.60	0.65
4					0.52	0.56
5						0.52

All the correlation was assessed using Spearman’s rho coefficient. All the results are statistically significant (*p* < 0.01)

IDAF-4C: index of dental anxiety and fear, T-MDAS: the Chinese version of the modified dental anxiety scale based on the Taiwanese sample

### Validity of T-MDAS

**Fig. 1:**
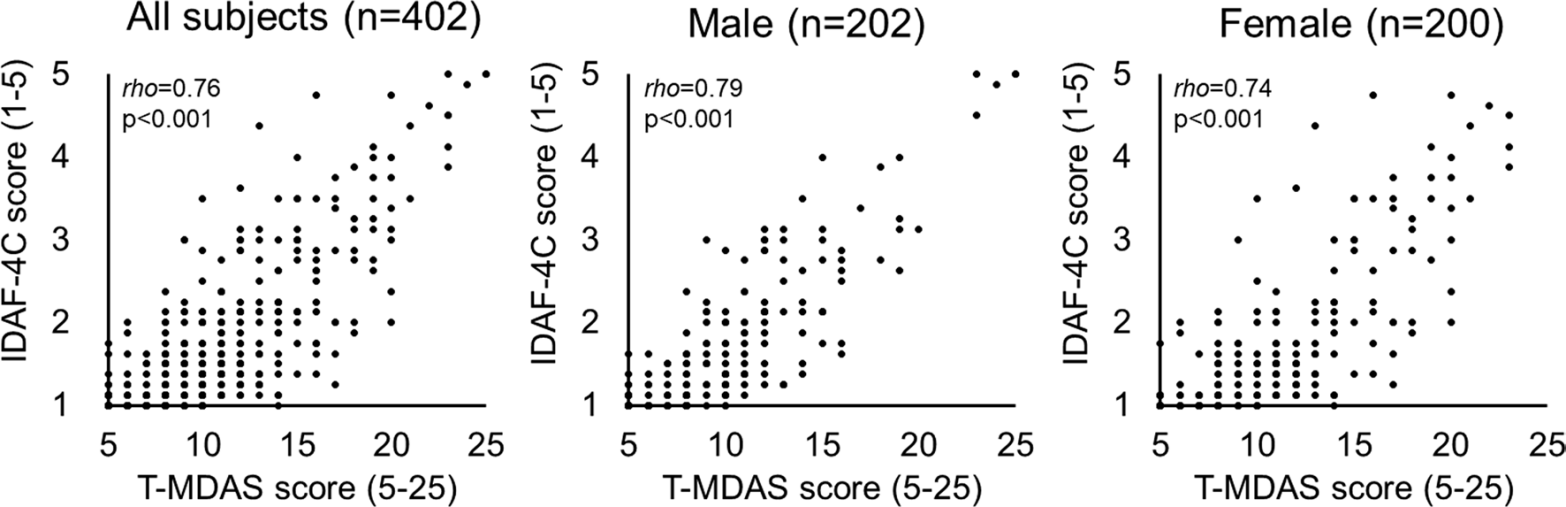
Association between the score of the Taiwan Modified Dental Anxiety Scale (T-MDAS) and the score of the index of dental anxiety and fear (IDAF-4C). The scores are significantly correlated for all the subjects and for the male and the female subgroup, respectively

**Fig. 2:**
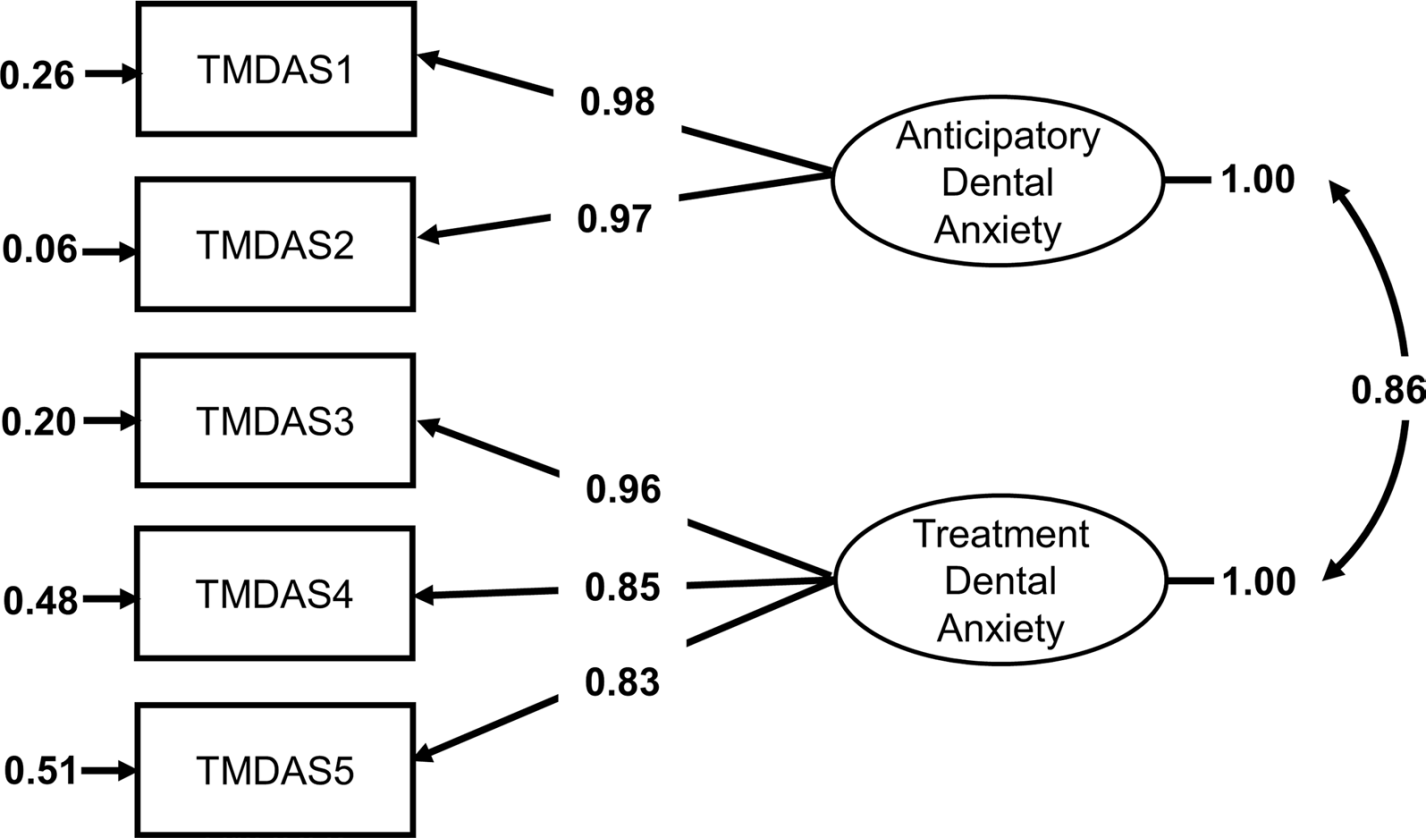
The path diagram of the confirmatory factor analysis. The results show that the T-MDAS score fits well to a two-factor model (anticipatory dental anxiety and treatment dental anxiety)

The T-MDAS scores were significantly correlated with the IDAF-4C scores (ρ = 0.76, *p* < 0.001) (Fig. 1). Moreover, the score from each of the five questions was significantly correlated with the IDAF-4C score, respectively (Table 3). The result is similar to that based on a Finnish sample (ρ = 0.74, [29]), suggesting good criterion-related validity. In addition, the correlation was statistically significant for both female and male subgroups (female: ρ = 0.74, *p* < 0.001; male: ρ = 0.79, *p* < 0.001) (Fig. 1). For discrimination validity, we found that subjects who regularly visited a dentist showed a lower T-MDAS score (mean = 10.1), compared to those who did not regularly visit a dentist (mean = 11.4) (two-tailed Mann–Whitney U test, *p* = 0.021). Consistently, the IDAF-4C assessment showed that subjects who regularly visited a dentist showed a lower IDAF-4C score (mean = 1.6), compared to those who did not regularly visit a dentist (mean = 2.0) (two-tailed Mann–Whitney U test, *p* < 0.001). The result supports for good discrimination validity of the T-MDAS. For construct validity, the CFA revealed that the data from the T-MDAS fit well to the two-factor model (*χ*^2^ = 10.5, *p* = 0.032, with RMSEA = 0.06, CFI = 0.99, GFI = 0.99 NFI = 0.99) (Fig. 2). The finding supports for good construct validity of the T-MDAS.

### Association between dental anxiety and dental-visiting behavior

**Fig. 3:**
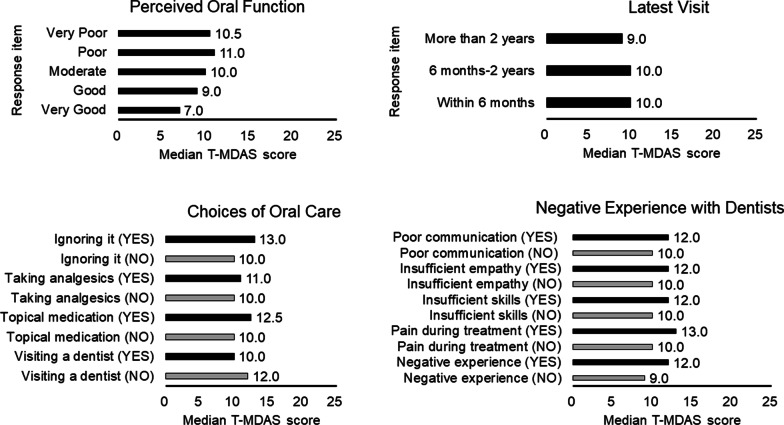
Association between the T-MDAS score and dental-visiting behavior and experience. The T-MDAS score significantly differs between different degrees of Perceived oral function, but not between the duration of Latest visit. The T-MDAS score significantly differs in the choices of oral care and negative experience with dentists in the subjects. Please note that the category ‘negative experience (NO)’ denotes the results that subjects responded ‘YES’ in the question ‘No negative experience’ and the category ‘negative experience (YES)’ denotes the results that subjects responded ‘NO’ in the question ‘no negative experience’. The modification is made to unify the direction of comparison across each item

For the variable Perceived Oral Function, subjects reporting different perception of their oral function showed a significant difference in the T-MDAS score (Kruskal Wallis test, *p* < 0.001). The subjects reporting a ‘Very Good’ function showed the lowest T-MDAS score (median = 7.0), while the subjects reporting a’Poor’ function showed the highest T-MDAS score (median = 11.0). For the variable Latest Visit, subjects who delayed a dental visit with different periods did not show a significant difference in the T-MDAS score (Kruskal Wallis test, *p* = 0.26) (Fig. 3).

For the variable Choices of Oral Care, the subjects who visited a dentist showed a lower T-MDAS score, compared to those who did not (Mann–Whitney U test, *p* = 0.001). In contrast, the subjects who used analgesic and just ignored it showed a higher T-MDAS score, compared to those who did not (Mann–Whitney U test, *p* = 0.038 and < 0.001, respectively). The T-MDAS score of the subjects who used topical medication and those who did not was not statistically significant (Mann–Whitney U test, *p* = 0.063). For the variable Negative Experience with Dentists, the subjects without negative experience showed a lower T-MDAS score, compared to those who did not (Mann–Whitney U test, *p* < 0.001). In contrast, the subjects with pain during treatment showed a higher T-MDAS score, compared to those who did not (Mann–Whitney U test, *p* < 0.001). The subjects who felt insufficient skills of dentists showed a higher T-MDAS score, compared to those who did not (Mann–Whitney U test, *p* = 0.010). The subjects who felt insufficient empathy of dentists showed a higher T-MDAS score, compared to those who did not (Mann–Whitney U test, *p* = 0.006). Finally, the subjects who felt poor communication with dentists showed a higher T-MDAS score, compared to those who did not, with a trend of statistical significance (Mann–Whitney U test, *p* = 0.056) (Fig. 3).

### The proportion of high-dental anxiety individuals

**Fig. 4:**
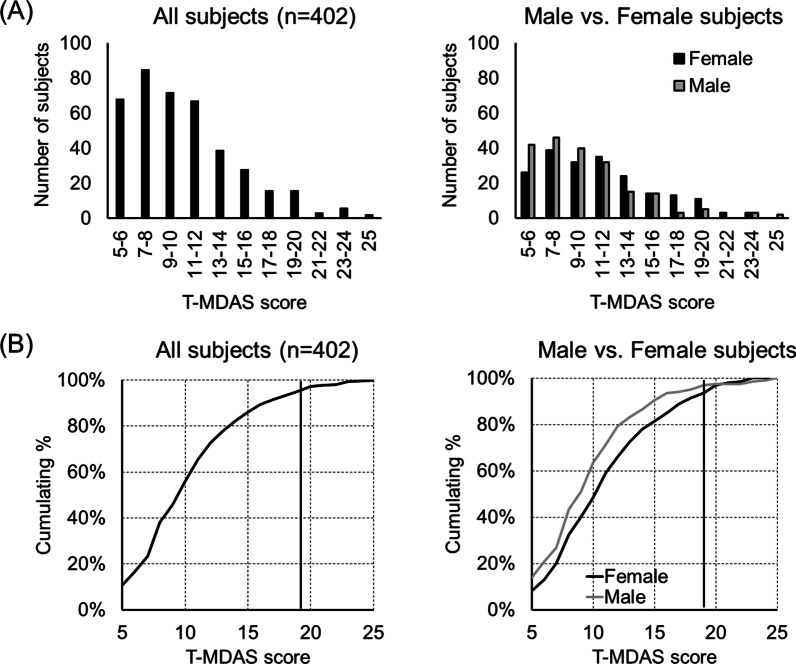
The proportion of high dental anxiety subjects. (**A**) The statistical distribution of the T-MDAS score for all the subjects and gender subgroups. All the distributions show a rightward-skewed pattern. (**B**) The cumulating distribution of the score reveals a higher proportion of high-dental anxiety subjects in the female subgroup, compared to the male subgroup

Figure 4A shows the distribution of the T-MDAS score for all subjects and the female and the male subgroups, respectively. The score distribution presents a rightward-skewed pattern, with the mode score at 7–8 points (Fig. 4A). The pattern suggests that while most subjects show lower dental anxiety, a few subjects show a great degree of dental anxiety. Notably, the male subgroup showed more subjects with a lower (i.e., 5–10) T-MDAS score and fewer subjects with a higher (i.e. > 11) T-MDAS score, compared to the female subgroup. The pattern of the distribution corresponds to the gender-related difference in the T-MDAS score (Table 2). We estimated the proportion of high-dental anxiety individuals, according to the cut-off value (19 points) established by previous studies [7, 31]. As shown in Fig. 4B, for all subjects, 6.7% of them reported the T-MDAS score ≧ 19. The proportion also differs between gender subgroups. The proportion of subjects with the T-MDAS score ≧ 19 was 8.5% and 5.0%, respectively for the female and the male subgroups (Fig. 4B).

## Discussion

### Major findings of the study

First, our results showed good criterion-related validity, discrimination validity, construct validity, internal consistency, and temporal stability for the T-MDAS, a Chinese version of the MDAS based on Taiwan adults. Second, we found that several behavioral factors were related to higher dental anxiety, including the lack of a regular dental visit, the use of analgesics for oral care, and ignorance of treatment, when subjects felt uncomfortable with their oral status. A poor perception of oral function and worse experience with dentists, including feeling pain during treatment, feeling insufficient skills and empathy of dentists, and poor patient-dentist communication, were associated with higher dental anxiety. Finally, we found that within our sample, 6.7% of the subjects showed high dental anxiety. The proportion differed between the female (8.5%) and the male (5.0%) subgroups.

### Association with dental anxiety and dental-visiting behavior and experience

By definition, dental anxiety focuses on ‘dental care-related’ anxiety [32]. Therefore, the factors intrinsic to dental treatment, such as the patient-dentist relationship [21], negative experience of treatment [22], and pain [2], have been conceived as the major factors contributing to dental anxiety and fear. Our findings revealed an association between dental fear and anxiety and these intrinsic factors similar to that reported by previous studies. For example, we found that subjects with a negative experience of dental treatment showed a higher T-MDAS score, compared to those who did not. In addition, subjects who perceived that dentists lack skills, empathy, and good communication with patients, showed a higher T-MDAS score, compared to those who did not. A recent study revealed that in the primary dental care services of the UK, patients’ anxiety of dental treatment was effectively reduced when dental staff performed anxiety screening for the patients [33]. Such an active engagement of a short questionnaire assessment, as part of good communication between patients and dental staff, may confer a beneficial effect for relieving patients’ anxiety [33]. Furthermore, results from a cross-sectional survey in the UK revealed that dental anxiety is associated with patients’ trust in dentists and their feelings of shame about their oral condition [34]. Consistently, our findings revealed that higher dental anxiety was associated with subjects’ perception of the lack of skills and empathy of dentists. The findings suggest that dental anxiety is highly associated with patients’ experience during treatment, in which patient-dentist interaction may play a key role.

### Gender-related difference in dental anxiety

Our result is consistent with the previous findings from different countries, which showed a higher MDAS score in female subjects, compared to male subjects (e.g., Turkey [35], China [8], Italy [15]). Consistently, clinical research revealed that female patients may report higher pre-procedural anxiety before receiving intra‐oral buccal mucosa biopsy [36] and extraction of horizontally impacted wisdom teeth [28], and medical procedures, such as gastroscopy [37]. Because pain of dental treatment is markedly associated with anxiety, a potential interpretation of the gender-related difference in dental anxiety is that female and male subjects differ in pain perception. Notably, while both genders may have similar pain threshold (e.g., heat pain stimuli at lips [38] and hands [39]), the willingness to report pain may differ due to the gender role (e.g. an expectation to be ‘macho’ for male individuals) [39]. Notably, the IDAF-4C score did not reveal a significant gender-related difference (two-tailed Mann–Whitney U test, *p* = 0.40) (Table 2). The gender-effect, as assessed using the IDAF-4C, is less clear-cut in the literature. For example, previous studies showed a significant gender-related difference in the summed IDAF-4C score from Australian population (female: 15.20, male: 13.56) [12], but insignificant difference in the mean IDAF-4C score from Finnish population (female: 1.49, male: 1.36) [29]. The discrepancy between the results from the MDAS and the results from the IDAF-4C may be accounted for by the questions from the assessments. In contrast to the T-MDAS that primarily focuses on the emotional aspects of dental anxiety (e.g., how anxious one feels), the IDAF-4C, additionally, focuses on the behavioral and cognitive aspects of anxiety (e.g., to delay making appointments or to expect something really bad) [25]. Therefore, the gender-related difference in emotional experience can be less weighted in the IDAF-4C, compared to that in the T-MDAS.

### Comparison between the current and previous findings

Notably, when setting the cut-off point of high-dental anxiety at 19 points, we found the proportion of high-dental anxiety individuals in our study is lower than that reported by previous studies, which adopted the same cut-off point (e.g., 6.8% for a clinical sample from the U.S. [9], 8.7% for a community sample from China [8], and 11.6% for a sample from the U.K. [31]). The difference in the proportion of high-dental anxiety individuals may be interpreted from several aspects. In addition to the ‘intrinsic factors’ that relate to dental treatment per se (e.g., pain and poor patient-dentist relationship), there would be some extrinsic factors contributing to dental fear, such as the approachability of dentists and the financial burden of receiving dental treatment. In Taiwan, most items of dental treatment, from preventive procedures (e.g., caries restoration and ultrasound scaling) to relatively invasive procedures (e.g., extraction of wisdom teeth and root canal treatment) are covered by the system of NHI. Therefore, patients may receive treatment without much financial burden. Meanwhile, there is a high density of private dental clinics in the urban area in Taiwan [40]. Therefore, the great approachability to dentists may contribute to the relatively lower proportion of high-dental anxiety individuals in our sample.

## Limitations of the current study

The findings of our study should be interpreted carefully with several limitations. First, we assessed the subjects both from community and clinical sites. However, both sites are located in the urban area in northern Taiwan. Therefore, the results may not fully represent the whole national population. Second, our results showed a strong association between dental anxiety and dental-visiting behavior and experience. However, as a cross-sectional and observational study, it is difficult to conclude the cause-effect relationship between dental anxiety, dental-visiting behavior, and the related experience. Thirdly, when evaluating the proportion of high-dental anxiety subjects, we followed the cut-off point based on a U.K. sample [6, 7] to compare our results with the previous findings using the same cut-off point. However, the cut-off point may not validly discriminate the clinical symptoms of high dental anxiety or dental phobia in Taiwan. Further research is required to establish a valid cut-off point for clinical usage.

## Conclusion

The T-MDAS is a valid tool for assessing adult dental anxiety. The score is highly associated with dental-visiting behavior and experience of dental patients.

## Supplementary Information

**Additional file 1**. The final version of the scale used in the study.

## Data Availability

The datasets generated during and analyzed during the current study are not publicly available due to regulations on the privacy of the subjects according to the guidelines from the local Internal Review Board but are available from the corresponding author on reasonable request.
