# Supplementary material for: Translation and validation of modified dental anxiety scale based on adult Taiwan population
Source: BMC Oral Health. 2021 Dec 17;21:647. doi: 10.1186/s12903-021-02017-w (PMC8684197; doi:10.1186/s12903-021-02017-w)
Supplement: Supplementary file 1 — Additional file 1. The final version of the scale used in the study. [file 12903_2021_2017_MOESM1_ESM.docx]

**說明：**

下列問題是關於您**牙科就診時**的**焦慮程度**調查。請針對每一個問題選擇最接近您感覺的答案，並勾選。

1. 如果你**明天要去看牙醫**，你的感覺如何？

🞎一點都不焦慮 🞎輕微焦慮 🞎有些焦慮 🞎很焦慮 🞎非常焦慮

1. 假如你正坐在牙科候診室裡，**等待接受治療**，你的感覺如何？

🞎一點都不焦慮 🞎輕微焦慮 🞎有些焦慮 🞎很焦慮 🞎非常焦慮

1. 假如你正準備要接受**牙齒鑽洞**，你的感覺如何？

🞎一點都不焦慮 🞎輕微焦慮 🞎有些焦慮 🞎很焦慮 🞎非常焦慮

1. 假如你正準備要接受**洗牙**，你的感覺如何？

🞎一點都不焦慮 🞎輕微焦慮 🞎有些焦慮 🞎很焦慮 🞎非常焦慮

1. 假如牙醫正準備對你上顎後牙的牙齦**施打麻藥**，你的感覺如何？

🞎一點都不焦慮 🞎輕微焦慮 🞎有些焦慮 🞎很焦慮 🞎非常焦慮
